# FDG: Five decades of transforming molecular imaging and still illuminating the brain

**DOI:** 10.1177/0271678X261465845

**Published:** 2026-06-23

**Authors:** Shokouh Arjmand, Paul Cumming, Albert Gjedde

**Affiliations:** 1 Department of Physiology and Pharmacology, Karolinska Institutet, Stockholm, Sweden; 2 Translational Neuropsychiatry Unit, Department of Clinical Medicine, Aarhus University, Aarhus, Denmark; 3 School of Psychology and Counselling, Queensland University of Technology, Brisbane, QLD, Australia; 4 Department of Neuroscience, Panum Institute, University of Copenhagen, Copenhagen, Denmark; 5 Department of Neurology and Neurosurgery, McGill University, Montreal, QC, Canada

**Keywords:** [^18^F]fluorodeoxyglucose, brain disorders, FDG–PET, neuroimaging, positron emission tomography

## Abstract

Over half a century after its introduction, [^18^F]fluorodeoxyglucose ([^18^F]FDG) remains a cornerstone of molecular imaging by positron emission tomography (PET). This commentary reflects on [^18^F]FDG–PET’s transformative role in neuroscience, highlighting its fundamental contributions to understanding brain metabolism and supporting clinical diagnoses of brain disorders, and the continuing evolution and refinement of brain PET imaging.

Following upon the synthesis of the glucose analog 2-deoxy-2-fluoro-D-glucose ([^19^F]FDG) by Pacák and Cerný at the Charles University in 1968,^1^ the year 2026 marks 50 years since the initial reports introducing [^18^F]fluorodeoxyglucose ([^18^F]FDG) for positron emission tomography (PET),^2,3^ which invites reflection upon one of the most transformative pillars of modern diagnostic and research medical imaging tools. The conceptual basis of [^18^F]FDG–PET quantitation of the metabolic rate for glucose derives from early studies of hepatic glucose clearance by Claude Bernard (1813–1878), and the combination of arterial-venous glucose concentration differences with perfusion data to calculate metabolic rates, as introduced by August Krogh (1874–1949). More immediately arising from the autoradiographic method with 2-deoxy-D-[^14^C]glucose developed by Louis Sokolof (1921–2015) and colleagues, molecular imaging with [^18^F]FDG leveraged the principle that cellular activity drives glucose demand. [^18^F]FDG–PET has allowed clinicians and researchers to bridge the bench-to-bedside gap, and to view specific disease processes not merely as static anatomical abnormalities, but as indicators of dynamic biological processes. Between 1980 and 2020, publication rates under the key words “FDG and brain” underwent exponential growth, now attaining a plateau of around 500 publications/year, versus around 3000 publications/year for mainly oncological [^18^F]FDG studies in peripheral organs. Indeed, no other radiopharmaceutical has had such a profound and sustained impact on fundamental neuroscience, and with translational impact across clinical disciplines, notably oncology, cardiology, psychiatry, and neurology.

Decades before the present era of connectomics, diverse PET tracers, and multimodal imaging, [^18^F]FDG–PET offered a window into the living brain’s metabolic activity, which was a revolutionary innovation. By enabling the mapping of cerebral glucose consumption in vivo, [^18^F]FDG–PET transformed the study of brain function and disease in a multidisciplinary approach,^4^ reshaping both clinical neurology and cognitive neuroscience, as exemplified by pioneering work on sensory activation.^5^ Because neuronal signaling is energetically demanding, regional [^18^F]FDG uptake is a robust indicator of neuronal and synaptic function. This principle enabled the first whole-brain functional mapping of awake humans,^6,7^ thereby grounding abstract concepts of cognition and behavior in measurable biological processes. Compartmental modeling analyses for the irreversible and reversible cerebral uptake of [^18^F]FDG have since generalized to thousands of enzyme substrates and receptor ligands for PET. Thus, in conjunction with studies of cerebral perfusion, [^18^F]FDG–PET laid the conceptual groundwork for molecular brain imaging.

In the simplest interpretation of the [^18^F]FDG–PET signal in brain, trapping reflects neuronal and synaptic activity. However, cell sorting techniques have shown considerable uptake in CD11b-positive microglia cells isolated from brain, which was augmented in association with amyloid pathology, whereas other such studies revealed an age-dependent increase in the proportion of astrocytic trapping in mouse brain. These results together show the extensive contribution of immunocompetent microglia to the brain’s glucose budget, and substantiate a model where glycolytic metabolism in astrocytes supports the glutamate–glutamine cycle and the transfer of lactate to adjacent neurons for oxidative metabolism. Furthermore, [^18^F]FDG–PET can give an indirect measure of alternate metabolite pathways in brain, as in the shift to ketone body metabolism.

Computerized tomography and subsequent structural MRI provide exquisite morphological detail, but do not interrogate brain function per se. As such, [^18^F]FDG–PET brought about a paradigm shift in clinical neurology, with a particularly enduring impact on the assessment of cognitive impairment and neurodegenerative disorders. Formerly, a definitive diagnosis of Alzheimer’s disease was incumbent upon postmortem analysis. [^18^F]FDG–PET now allows clinician scientists to differentiate with unprecedented accuracy Alzheimer’s disease from frontotemporal dementia, dementia with Lewy bodies, or the recently identified disease entity limbic-predominant age-related TDP-43 encephalopathy (LATE), based on their characteristic patterns of regional hypometabolism.^8^ Statistical mapping of [^18^F]FDG uptake (with normalization to some reference region) enables the discrimination of neurodegenerative conditions with high diagnostic performance, and global normalization reveals patterns of relative metabolic increases and decreases that are predictive of disease progression. By reducing misdiagnosis, such success in differential diagnosis and prognostication have fundamentally altered the management of cognitively impaired patients and became a critical tool for accurately stratifying patients in clinical trials.

The new era in molecular imaging for clinical neurology dominated by highly specific molecular targets such as β-amyloid, tau, and synaptic vesicle glycoprotein 2A has hardly rendered obsolete [^18^F]FDG–PET, which retains its status as the paramount marker of neurodegeneration and functional impairment. [^18^F]FDG–PET anchors clinical symptoms to metabolic changes, as distinct from pathology markers such as plaques, tangles, and synaptic density.

In recent applications, artificial intelligence and machine learning enhance the sensitivity of [^18^F]FDG–PET for early and discriminative diagnosis of Alzheimer’s disease,^9^ potentially identifying subtle metabolic declines that precede by years the onset of structural atrophy or overt cognitive deficits. Recently-developed functional imaging methods with [^18^F]FDG make possible the detection of rapid and transient changes in tracer delivery and metabolism, rather than requiring the assumption of a prolonged steady-state during tracer uptake. Hybrid [^18^F]FDG–PET/fMRI studies are enabling a deeper integration of cerebral metabolism with functional connectivity in diverse pathophysiological contexts. The gold standard of [^18^F]FDG–PET quantitation long required serial sampling of arterial blood, but long axial field (LAFOV) tomographs can provide an image-derived input function from large vessels such as the aortic arch.^10^ This should facilitate a second phase of quantitative PET, which had been largely abandoned due to the impracticality of drawing arterial blood samples in patient populations. LAFOV [^18^F]FDG–PET also makes possible consideration of the whole-body metabolic connectome, which may prove to reveal new insights into the relationship between cerebral and peripheral metabolism; many neuropsychiatric disorders probably entail body-wide disturbances. Finally, high-sampling rate [^18^F]FDG–PET acquisitions along with an IDIF has the potential to enable mapping of cerebral blood flow, [^18^F]FDG extraction fraction, and permeability surface area product, as well as the usual influx rates, all in the setting of a single recording. Additional references supporting this work are provided in the supplemental material.

Half a century on, [^18^F]FDG continues to illuminate the metabolic foundations of the human brain and its functions. Its legacy extends beyond transforming the brain from an inaccessible organ into a measurable and dynamic system. Through its critical role in characterizing disease trajectories and accelerating the development of targeted therapies, as well as guiding complex surgical interventions such as in intractable epilepsy, and enabling earlier biological and diagnostic clarity, [^18^F]FDG–PET translates research innovations into profoundly improved biological insight. Few scientific innovations can claim equally lasting global impacts, while continuing to drive modern translational neuroscience.

## Supplemental Material

sj-docx-1-jcb-10.1177_0271678X261465845 – Supplemental material for FDG: Five decades of transforming molecular imaging and still illuminating the brain Supplemental material, sj-docx-1-jcb-10.1177_0271678X261465845 for FDG: Five decades of transforming molecular imaging and still illuminating the brain by Shokouh Arjmand, Paul Cumming and Albert Gjedde in Journal of Cerebral Blood Flow & Metabolism
