# Supplementary material for: FDG: Five decades of transforming molecular imaging and still illuminating the brain
Source: J Cereb Blood Flow Metab. 2026 Jun 23:0271678X261465845. Online ahead of print. doi: 10.1177/0271678X261465845 (PMC13368769; doi:10.1177/0271678X261465845)
Supplement: sj-docx-1-jcb-10.1177_0271678X261465845 – Supplemental material for FDG: Five decades of transforming molecular imaging and still illuminating the brain [file sj-docx-1-jcb-10.1177_0271678X261465845.docx]

Supplemental material

**FDG: Five Decades of Transforming Molecular Imaging and Still Illuminating the Brain**

Shokouh Arjmand ^1,2^, Paul Cumming ^3^, Albert Gjedde ^2,4,5^

^1^ Department of Physiology and Pharmacology, Karolinska Institutet, Stockholm, Sweden.

^2^ Translational Neuropsychiatry Unit, Department of Clinical Medicine, Aarhus University, Aarhus, Denmark

^3^ School of Psychology and Counselling, Queensland University of Technology, Brisbane, Australia

^4^ Department of Neuroscience, University of Copenhagen, Panum Institute, Copenhagen, Denmark

^5^ Department of Neurology and Neurosurgery, McGill University, Montreal, Québec, Canada

Correspondence to: albert.gjedde@clin.au.dk (A. Gjedde) and shokouh@clin.au.dk (S. Arjmand)

Translational Neuropsychiatry Unit, Department of Clinical Medicine, Aarhus University, Palle Juul-Jensens Boulevard 99, A601/A701, Aarhus N 8200, Denmark.

This supplementary material contains the expanded bibliography for the commentary. ^9–21^

1 Bartos LM, Kunte ST, Beumers P, Xiang X, Wind K, Ziegler S *et al.* Single-Cell Radiotracer Allocation via Immunomagnetic Sorting to Disentangle PET Signals at Cellular Resolution. *J Nucl Med* 2022; **63**: 1459–1462.

2 Bartos LM, Kunte ST, Wagner S, Beumers P, Schaefer R, Zatcepin A *et al.* Astroglial glucose uptake determines brain FDG-PET alterations and metabolic connectivity during healthy aging in mice. *Neuroimage* 2024; **300**. doi:10.1016/J.NEUROIMAGE.2024.120860.

3 Li X, Young AJ, Shi Z, Byanyima J, Vesslee S, Reddy R *et al.* Pharmacokinetic effects of a single dose nutritional ketone ester supplement on brain glucose and ketone metabolism in alcohol use disorder. *Psychiatry Res Neuroimaging* 2026; **357**: 112154.

4 Ford JN, Sweeney EM, Skafida M, Glynn S, Amoashiy M, Lange DJ *et al.* Heuristic scoring method utilizing FDG-PET statistical parametric mapping in the evaluation of suspected Alzheimer disease and frontotemporal lobar degeneration. *Am J Nucl Med Mol Imaging* 2021; **11**: 313.

5 Perovnik M, Tang CC, Namías M, Eidelberg D. Longitudinal changes in metabolic network activity in early Alzheimer’s disease. *Alzheimers Dement* 2023; **19**: 4061–4072.

6 Hahn A, Falb P, Murgas M, Klug S, Reed MB, Godbersen GM *et al.* EXPRESS: 18F]FDG functional PET revisited: a new perspective on the temporal dynamics of brain glucose metabolism. *Journal of Cerebral Blood Flow & Metabolism* 2026. doi:10.1177/0271678X261455750.

7 Geist BK, Guthrie J, Kertesz H, Kiefer FW, Kulterer OC, Nakuz T *et al.* The metabolic organ connectome: A novel approach to measure allostatic load during health-to-disease transition. *Med (N Y)* 2025; **6**. doi:10.1016/J.MEDJ.2025.100881.

8 Chung KJ, Chaudhari AJ, Nardo L, Jones T, Chen MS, Badawi RD *et al.* Quantitative Total-Body Imaging of Blood Flow with High-Temporal-Resolution Early Dynamic 18F-FDG PET Kinetic Modeling. *J Nucl Med* 2025; **66**: 973–980.

9 Shivamurthy VKN, Tahari AK, Marcus C, Subramaniam RM, Shivamurthy VKN, Tahari AK *et al.* Brain FDG PET and the Diagnosis of Dementia. *https://www.ajronline.org/* 2014; **204**: W76–W85.

10 Minoshima S, Mosci K, Cross D, Thientunyakit T. Brain [F-18]FDG PET for Clinical Dementia Workup: Differential Diagnosis of Alzheimer’s Disease and Other Types of Dementing Disorders. *Semin Nucl Med* 2021; **51**: 230–240.

11 Gjerum L, Frederiksen KS, Henriksen OM, Law I, Bruun M, Simonsen AH *et al.* Evaluating 2-[18F]FDG-PET in differential diagnosis of dementia using a data-driven decision model. *Neuroimage Clin* 2020; **27**: 102267.

12 Gjedde A, Wienhard K, Heiss WD, Kloster G, Diemer NH, Herholz K *et al.* Comparative regional analysis of 2-fluorodeoxyglucose and methylglucose uptake in brain of four stroke patients. With special reference to the regional estimation of the lumped constant. *J Cereb Blood Flow Metab* 1985; **5**: 163–178.

13 Gjedde A. Calculation of cerebral glucose phosphorylation from brain uptake of glucose analogs in vivo: a re-examination. *Brain Res* 1982; **257**: 237–274.

14 Chung KJ, Abdelhafez YG, Spencer BA, Jones T, Tran Q, Nardo L *et al.* Quantitative PET imaging and modeling of molecular blood-brain barrier permeability. *medRxiv* 2024. doi:10.1101/2024.07.26.24311027.

15 Smith CLC, Zwezerijnen GJC, den Hollander ME, Greuter HNJM, Gerards NR, Zijlstra J *et al.* Validating image-derived input functions of dynamic 18F-FDG long axial field-of-view PET/CT studies. *Frontiers in Nuclear Medicine* 2025; **5**: 1556848.

16 Labarthe A, Varet S, Savale L, Montani D, Humbert M, Faure S *et al.* Personalized mapping of body homeostasis using whole-body PET connectomics and routine FDG PET imaging. *Communications Medicine 2026* 2026. doi:10.1038/s43856-026-01549-y.

17 Del Sole A, Malaspina S, Magenta Biasina A. Magnetic resonance imaging and positron emission tomography in the diagnosis of neurodegenerative dementias. *Funct Neurol* 2017; **31**: 205.

18 Portnow LH, Vaillancourt DE, Okun MS. The history of cerebral PET scanning. *Neurology* 2013; **80**: 952–956.

19 Alavi A, Reivich M. Guest editorial: The conception of FDG-PET imaging. *Semin Nucl Med* 2002; **32**: 2–5.

20 Jones T, Rabiner EA. The development, past achievements, and future directions of brain PET. *Journal of Cerebral Blood Flow and Metabolism* 2012; **32**: 1426–1454.

21 Kishore N, Goel N. Deep learning based diagnosis of Alzheimer’s disease using FDG-PET images. *Neurosci Lett* 2023; **817**: 137530.
